# Efficient Implementation of NIST LWC ESTATE Algorithm Using OpenCL and Web Assembly for Secure Communication in Edge Computing Environment

**DOI:** 10.3390/s21061987

**Published:** 2021-03-11

**Authors:** BoSun Park, Seog Chung Seo

**Affiliations:** Department of Financial Information Security, Kookmin University, Seoul 02707, Korea; 20175206@kookmin.ac.kr

**Keywords:** web, Web Assembly, OpenCL, LWC, fast implementation

## Abstract

In edge computing service, edge devices collect data from a number of embedded devices, like sensors, CCTVs (Closed-circuit Television), and so on, and communicate with application servers. Since a large portion of communication in edge computing services are conducted in wireless, the transmitted data needs to be properly encrypted. Furthermore, the application servers (resp. edge devices) are responsible for encrypting or decrypting a large amount of data from edge devices (resp. terminal devices), the cryptographic operation needs to be optimized on both server side and edge device side. Actually, the confidentiality and integrity of data are essential for secure communication. In this paper, we present two versions of security software which can be used on edge device side and server side for secure communication between them in edge computing environment. Our softwares are basically web-based application because of its universality where the softwares can be executed on any web browsers. Our softwares make use of ESTATE (Energy efficient and Single-state Tweakable block cipher based MAC-Then-Encrypt)algorithm, which is a promising candidate of NIST LWC (National Institute of Standards and Technology LightWeight Cryptography) competition and it provides not only data confidentiality but also data authentication. It also implements the ESTATE algorithm using Web Assembly for efficient use on edge devices, and optimizes the performance of the algorithm using the properties of the underlying block cipher. Several methods are applied to efficiently operate the ESTATE algorithm. We use conditional statements to XOR the extended tweak values during the operation of the ESTATE algorithm. To eliminate this unnecessary process, we use a method of expanding and storing the tweak value through pre-computation. The measured results of the ESTATE algorithm implemented with Web Assembly and the reference C/C++ ESTATE algorithm are compared. ESTATE implemented as Web Assembly is measured in web browsers Chrome, FireFox, and Microsoft Edge. For efficiency on server side, we make use of OpenCL which is parallel computing framework in order to process a number of data simultaneously. In addition, when implementing with OpenCL, using conditional statements causes performance degradation. We eliminated the conditional statement using the loop unrolling method to eliminate the performance degradation. In addition, OpenCL operates by moving the data to be encrypted to the local memory because the local memory has a high operation speed. TweAES-128 and TweAES-128-6, which have the same structure as AES algorithm, can apply the previously existing studied T-table method. In addition, the input value 16-byte is processed in parallel and calculated. In addition, since it may be vulnerable to cache-timing attack, it is safely operated by applying the previously existing studied T-table shuffling method. Our softwares cover the necessary security service from edge devices to servers in edge computing services and they can be easily used for various types of edge computing devices because they are all web-based applications.

## 1. Introduction

Existing cloud computing methods provide overall services, such as data processing and transmission in servers and data centers. However, with the increase of users using cloud computing services, the amount of data that has to be processed by the server has increased. So, there is a system load in the process of data processing and transmission. To solve this problem, an edge computing method was created. Edge computing method is a method that processes data in devices, such as smartphones, unlike the method in which servers and data centers handle all services. Edge computing method reduces the system load because it only processes data generated by the device. It is also relatively efficient compared to cloud computing because it collects and processes data on its own. In the case of cloud computing, if a server is paralyzed, it is a fatal blow, but, because edge computing performs its own computing, it can effectively respond to failures. Therefore, we propose a web-based application edge computing method using Web Assembly. The existing edge computing method provides services by processing data sent from a server using a method optimized for hardware, such as ARM, RISC-V, and AVR. However, when edge computing is used using a variety of hardware, there is a disadvantage of having to implement a service and cryptographic algorithm according to each hardware. However, this method can be used generally in PCs (Personal Computer), smartphones, and IoT (Internet of Things) devices that can use web-based applications, such as web browsers and web apps. In addition, there is an advantage that can be used in common in various web-based applications without additional modification on implementation.

In addition, in edge computing method, communication between server and edge computing, communication between edge computing and users, and communication between edge computing will be achieved. For secure data communication, it is necessary to encrypt data and verify that the transmitted data is transmitted without change. So, encryption algorithm and authentication algorithm must be used separately. However, we use the LWC ESTATE (LightWeight Cryptography Energy efficient and Single-stateTweakable block cipher based MAC-Then-Encrypt) algorithm, which can do this process at once. In addition, it provides edge computing service by implementing encryption and authentication service of ESTATE algorithm with Web Assembly, which has better performance than JavaScript for communication using web-based applications.

We propose an efficient implementation of the ESTATE algorithm that uses OpenCL parallel processing to efficiently transfer data through the ESTATE algorithm as a web-based application that provides edge computing services on the server. Even if the server system load is reduced due to the edge computing method, the final processed data is stored on the server. It is a web-based application that provides edge computing services on the server and needs to transfer data using the ESTATE algorithm. Therefore, there is a need for a way to efficiently operate the ESTATE algorithm on the server. Therefore, we applied several additional methods to ensure that the ESTATE algorithm works efficiently for each environment.

### Contribution

The contribution of this paper is as follows:

1 Web-based application edge computing method using Web Assembly
  As the number of users using cloud computing services increases, so does the amount of data that must be processed. So, there is a system load in the process of providing the service. So, the edge computing approach was created. The edge computing method transmits and processes data to hardware, such as ARM, RISC-V, and AVR, to reduce system load. However, this method has the disadvantage of having to implement the service differently using each hardware environment and programming language. So, we propose a web-based application edge computing method using Web Assembly. Web Assembly was created to show similar performance to a low-level language. The web-based application edge computing method has the advantage that it can be used in common without additional modification in PCs, smartphones, and IoT devices that can use web-based applications. In addition, the edge computing method communicates data between server and web-base application, web-base application and user, and web-base application. So, the ESTATE algorithm that can generate the encryption process and tag for authentication at once is implemented using Web Assembly to provide edge computing services. Check how far Web Assembly has caught up with the low-level language in terms of performance. Web Assembly was run on Chrome, FireFox, and Microsoft Edge. At Chrome, FireFox, and Microsoft Edge, Web Assembly is approximately 11%, 10%, 9% slower than Reference C code, TweAES-128-6 is about 5%, 2%, 6% slower, and TweGIFT-128 is about 22%, 54%, and 17% slower than Reference C code.
2 LWC ESTATE parallel processing using OpenCL
  ESTATE (Energy efficient and Single-state Tweakable block cipher based MAC-Then-Encrypt) algorithm is designed to be used in a limited environment, but the data are finally stored on the server. Therefore, ESTATE algorithm optimization is also required in the server. ESTATE algorithm divides AD (Associated Data) and messages into 128-bit blocks, encrypts them one block at a time, and affects the next process, so it cannot process a large amount of data through parallel processing at once. Servers have to send data to multiple platforms, so if they are processed sequentially, the communication speed becomes slow. So, we propose a method of simultaneously generating multiple ciphertexts and tags to be sent to multiple web-based applications for edge computing using OpenCL parallel processing. As a result, the implemented TweAES-128, TweAES-128-6, and TweGIFT-128 implemented in OpenCL showed performance improvement of 6.69×, 7.31×, and 1.47×, respectively, compared to the reference C code.
3 Optimization method for safe and efficient operation of ESTATE algorithm
  ESTATE algorithm uses TweAES-128, TweAES-128-6, and TweGIFT-128. We propose several methods for safe and efficient operation, and apply the previously existing studied methods. In the operation process of TweAES-128, TweAES-128-6, and TweGIFT-128, there is a process of XOR operation by expanding the 4-bit tweak value. TweAES-128 and TweAES-128-6 expand to 8-bit, and TweGIFT-128 expand to 32-bit. However, only 0∼7, 15 are used as 4-bit tweak values. Therefore, we propose a way to store and use 8-bit, 32-bit extended tweak values for 94-bit tweak values through pre-computation. In the OpenCL implementations of TweAES-128, TweAES-128-6, and TweGIFT-128, to remove the performance load, we use a loop unwind method to remove the load and implement it using local memory with a relatively fast working speed. The operation process of TweAES-128 and TweAES-128-6 is the same as AES algorithm. Therefore, the T-table method, which was previously existing studied, was applied. In addition, AES algorithm is vulnerable to cache-timing attack, and TweAES-128 and TweAES-128-6 with the same structure will be vulnerable. Therefore, TweAES-128 and TweAES-128-6 are safely operated by applying the T-table shuffling method, which is the method previously existing studied. TweAES-128 and TweAES-128-6, which applied the table shuffling method previously existing studied, show about 7% and 51% performance overhead, respectively. It simply shuffles the T-table, so it shows little performance overhead.

The remainder of this paper is organized as follows. Section 2 provides a basic overview of the web environment, Web Assembly, OpenCL, Edge computing, and LWC ESTATE. Section 3 describes the relate work of OpenCL and Web Assembly. Section 4 describes the method proposed in the paper. Section 5 describes the performance measurement results. Finally, Section 6 concludes the paper.

## 2. Background

### 2.1. Edge Computing

**Figure 1:**
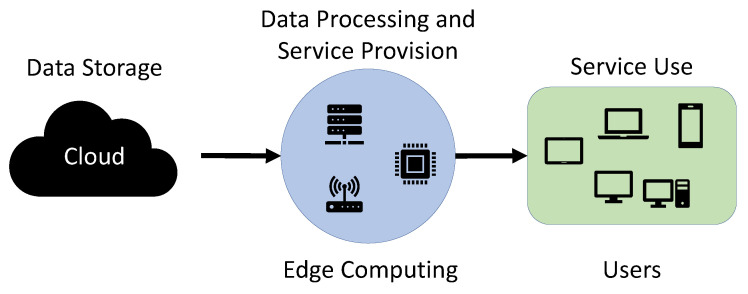
Edge computing structure.

Several companies have used cloud computing methods [1] to provide computing services, such as servers, storage, software, and analytics, over the Internet. Cloud computing is a method focused on centralizing services to several large data centers. However, such cloud computing also begins to have problems. As the number of people using cloud services increases, the amount of data processed by servers and data centers increases, and data delays occur in the process of analyzing and transmitting collected data. That is why edge computing [2,3,4] was created to solve problems, such as data processing speed, capacity, and security. Edge computing is performing computing at or near the physical location of a user or data source. In the case of cloud computing, data is processed in the data center, whereas edge computing is a method of processing data in devices, such as smartphones. Edge computing method has several advantages. When using cloud computing, the larger the amount of data to be processed, the higher the system load, but in the case of edge computing, data load can be reduced because only data generated by the device is processed. In addition, cloud computing has to strengthen security from the process of data transmission and delivery with a central server architecture, but edge computing is relatively more secure than cloud computing because it collects and processes data on its own. In addition, when the server is paralyzed when using cloud computing, the overall damage is seriously affected, but when using edge computing, it can effectively respond to failures because it performs its own computing. Figure 1 is the structure of the edge computing method. It shows a structure that does not process data in a server or data center but sends data to peripheral devices that will perform edge computing services and sends data to the user’s device after processing.

### 2.2. Web Environment

**Figure 2:**
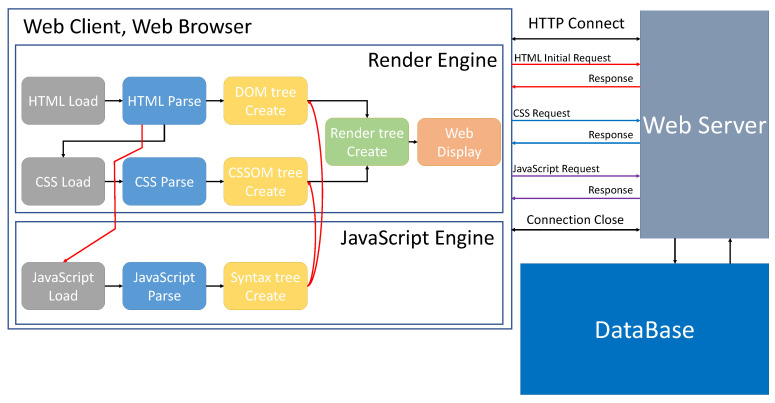
Web operation process [6].

Due to the continuous development of the web environment, various functions are being performed in the web environment. Due to the advancement of web technology, information on the web is displayed the same on different platforms to which networks are connected, such as PCs or smart devices. Web-based applications run within a web browser without communicating with the operating system. Due to the development of internet technology, and hardware performance improve, web technologies and libraries are continuously being created so that more complex and heavy calculations and functions can be made in a web environment. There are various web browsers in which these functions can be used, and various web browsers, such as Chrome, FireFox, and Microsoft Edge, exist. Each web browser has a JavaScript engine that renders JavaScript code and a rendering engine that provides visual services to users through web screens. Chrome uses V8 and Blink, FireFox uses SpiderMonkey and Gecko, and Microsoft Edge uses Chakra and EdgeHTML as JavaScript engines and rendering engines. There is Node.js [5], a software platform used for network application development. Node.js includes a built-in http server library, so it can be operated without additional software on the web server, and through this, more control over the operation of the web server is possible. In addition, web socket communication is possible using Node.js. Figure 2 shows the process of communication between the user who uses the web and the web server, and the process of storing data generated while using the web in the database.

### 2.3. Web Assembly

**Figure 3:**
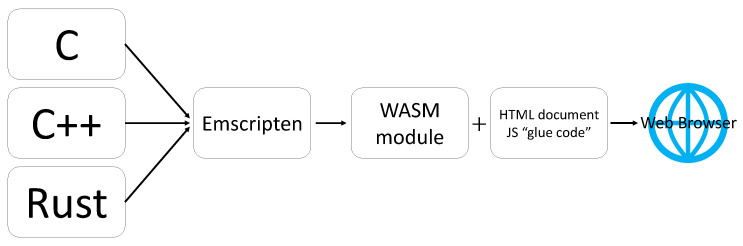
Web Assembly conversion process [6].

With the development of web-based applications, various organizations, companies, and individuals develop web-based applications to provide various services, and various web technologies are being developed. In addition, many users access web-based applications to use the various services and functions provided. These web-based applications are mainly developed in JavaScript, which is a cross-platform language, to display the same information to users on multiple platforms. However, as the number of web users increases, the amount of data that needs to be collected and processed increases, so it is important to increase the speed of processing data to reduce the load. In order to compute faster even in the web environment, Web Assembly [7,8] was created and it is constantly evolving. In addition, Web Assembly can be used by converting to languages with data types, such as C [9], C++ [10], and Typescript [11]. Therefore, it is possible to use previously implemented codes without additional modification. Due to the existence of data types, unlike JavaScript, mathematical operations allow the desired value to be computed without additional computation. Figure 3 shows the process of converting to Web Assembly using programming languages that have data types, such as C, C++, and Rust [12].

### 2.4. OpenCL

**Figure 4:**
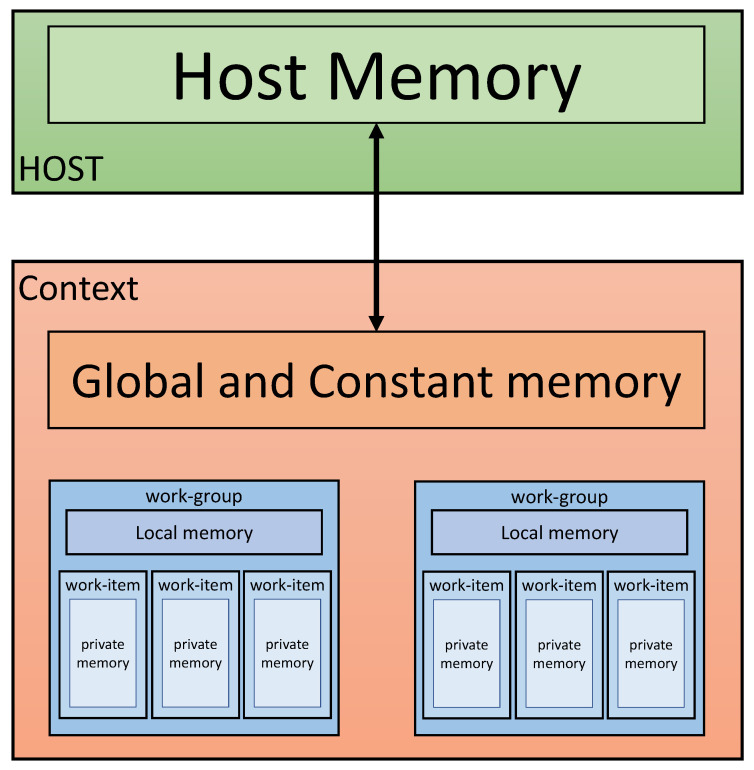
OpenCL memory structure [13].

OpenCL is an open general purpose parallel computing framework for creating programs that run on heterogeneous platforms, such as CPUs and GPUs. OpenCL provides task-based, data-based parallel computing. OpenCL can be used in AMD, Intel CPU, Intel integrated graphics, and NVIDIA graphics card products. Table 1 and Figure 4 show the four types of memory used in OpenCL and their respective functions.

**Figure 5:**
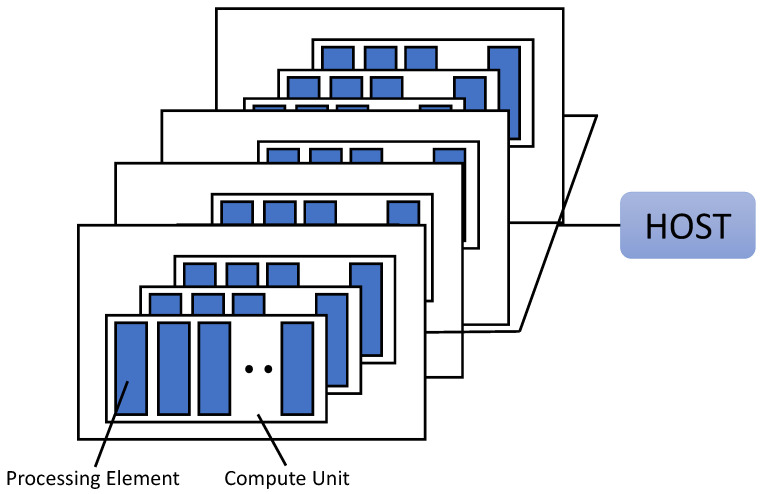
OpenCL platform model [13].

Figure 5 shows the OpenCL platform model consisting of one host and one or more devices. The OpenCL platform always contains only one host. Each device has one or more compute units, and each computational unit has one or more processing element (PE). The device is where the kernel runs. Devices are provided by CPU, GPU, DSP, and hardware manufacturers. And the actual calculation for the device is done in PE.

**Table 1:** OpenCL memory characteristics [14,15].

Memory	Characteristics
Global Memory	(1) Read and write from all work items (2) Placed in device’s main memory
Constant Memory	(1) Read only from all work items (2) Placed in device’s main memory
Local Memory	(1) Can be shared and used among work items in a work group (2) In many cases, a shared memory disposed on each operation unit is used.
Private Memory	(1) Dedicated memory area for work items (2) Often times you use registers used by processing elements.

### 2.5. Lightweight Cryptography (Lwc) Estate

Table 2 is a table of notations, operations, and algorithms used in the ESTATE algorithm.

Energy efficient and Single-state Tweakable block cipher-based MAC-Then-Encrypt (ESTATE) [16], one of the second round candidates for lightweight encryption algorithms, adopts FCBC-like [17] authentication and is a tunable block cipher-based authentication encryption system using OFB [18] encryption. ESTATE is based on the MAC-then-Encrypt paradigm [19]. ESTATE does not require field multiplications and has single-state, inverse-free, and RUP secure construction features [16]. In addition, ESTATE is divided into ESTATE mode and sESTATE mode. In ESTATE mode, TweAES-128 and TweGIFT-128 are proposed and used as core algorithms. TweAES-128 and TweGIFT-128 are modified versions of AES-128 [20] and GIFT-128 [21], respectively.

(1) $$\forall X\in \overset{n}{\underset{m=1}{⋃}}{0,1}^{m},X\mapsto \left\{\begin{array}{cc} 0^{n-\left|X\right|-1}∥1∥X & if\left|X\right|<n,\\ X & otherwise, \end{array}\right.$$

(2) $$\left(E_{1};E_{2}\right)?a:b:c:d:=\left\{\begin{array}{cc} a & ifE_{1}\wedge E_{2}\\ b & ifE_{1}\wedge \neg E_{2}\\ c & if\neg E_{1}\wedge E_{2}\\ d & if\neg E_{1}\wedge \neg E_{2} \end{array}.\right.$$

If the last block of the message and AD is smaller than 128-bit, padding is performed using Equation (1). Equation (2) is to determine the tweak value used in the ESTATE encryption process.

**Figure 6:**
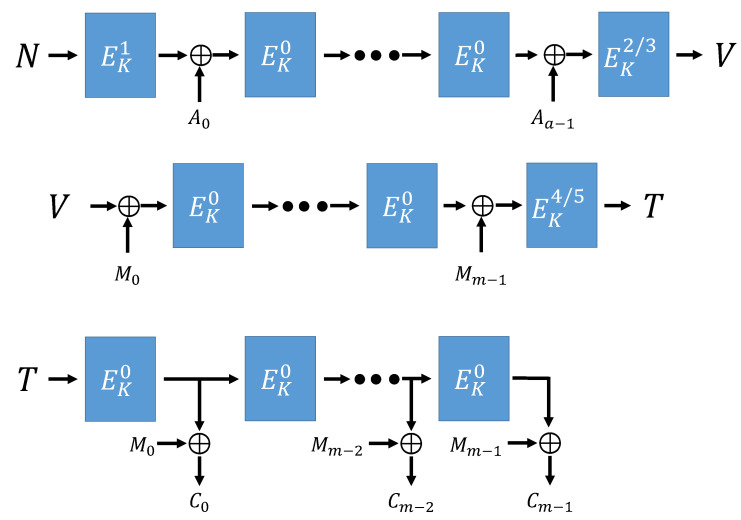
ESTATE mode (Using AD and Message) [16].

**Figure 7:**
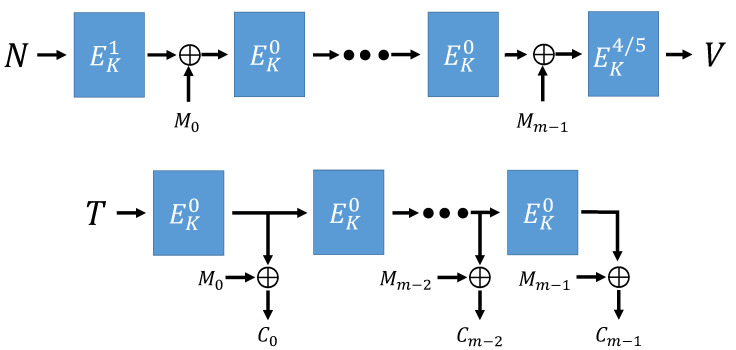
ESTATE mode (no AD, using Message) [16].

**Figure 8:**
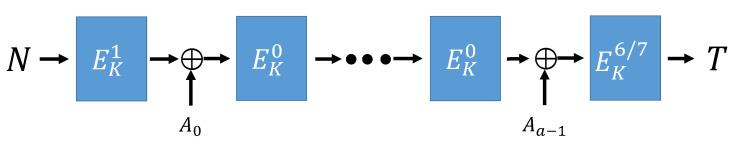
ESTATE mode (using AD, no Message) [16].

Algorithm 1 is the functions used in the overall encryption and decryption operation in ESTATE mode. MAC function [16] is a function that creates a tag. The FCBC^*^ [16] function is a function that determines the tweak value according to AD, message length, and encryption process conditions. OFB function [16] is a function that creates a cipher text using the created Tag value. Figure 6, Figure 7 and Figure 8 show ESTATE mode operation process when both AD and Message are used as input values, when only Message is used, and when only AD is used.

**Figure 9:**
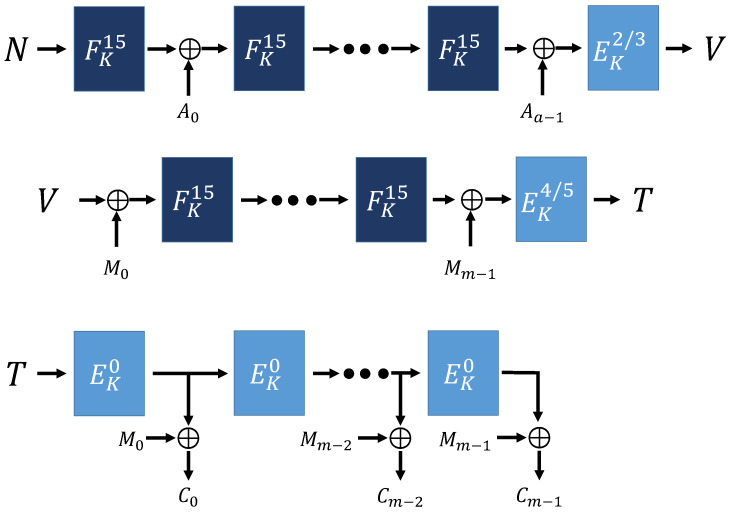
sESTATE mode (using AD and Message) [16].

**Figure 10:**
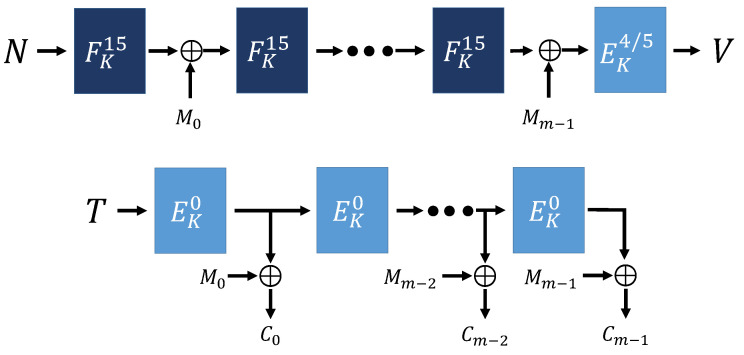
sESTATE mode (no AD, using Message) [16].

**Figure 11:**
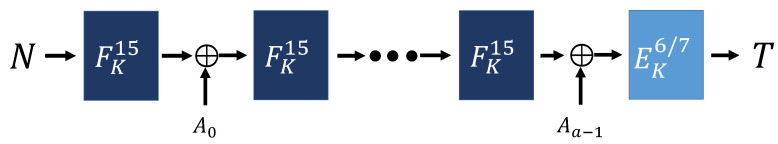
sESTATE mode (using AD, no Message) [16].

Algorithm 2 is the overall operation process of sESTATE mode. sESTATE mode has the same operation process as ESTATE mode. Figure 9, Figure 10 and Figure 11 show the process of sESTATE mode operation.
**Algorithm 1** ESTATE Encryption, Tag Creation, Authentication, and Decryption Algorithm [16]. 1: function ESTATE.ENC[$\tilde{E}$](K,N,A,M)18: function ESTATE.DEC[$\tilde{E}$](K,N,A,C,T)2:    $T\leftarrow MAC\left[\tilde{E}\right]\left(K,N,A,M\right)$19:    $M\leftarrow OFB\left[\tilde{E}\right]\left(K,T,C\right)$3:    $C\leftarrow OFB\left[\tilde{E}\right]\left(K,T,M\right)$20:    $T^{\prime }\leftarrow MAC\left[\tilde{E}\right]\left(K,N,A,M\right)$4:    retrun (C,T)21:    return $\left(T^{\prime }=T\right)$ ? *M* : ⊥ 5: function MAC[$\tilde{E}$](K,N,A,M)22: function FCBC${}^{*}\left[\tilde{E}\right]\left(K,T,D,t\right)$6:    if $\left|A\right|=0$ and $\left|M\right|=0$ then23:    $D_{d-1}∥\cdots ∥D_{0}\leftarrow D$7:       return $T\leftarrow {\tilde{E}}_{K}^{8}\left(N\right)$24:    for i=0 to d−2 do8:    $T\leftarrow {\tilde{E}}_{K}^{1}\left(N\right)$25:       $T\leftarrow {\tilde{E}}_{K}^{0}\left(T⊕D_{i}\right)$9:    if $\left|A\right|>0$ then26:    $T\leftarrow {\tilde{E}}_{K}^{t}\left(T⊕\mathrm{opt}\left(Dd-1\right)\right)$10:       $A_{a-1}∥\cdots ∥A_{0}\leftarrow A$27:    return *T*11:       $t\leftarrow (\left|M\right|>0$ ; $\left|A_{a-1}\right|=n)$ ? 2 : 3 : 6 : 7
12:       T← FCBC${}^{*}\left[\tilde{E}\right]\left(K,T,M,t\right)$28: function OFB[$\tilde{E}$](K,T,M)13:    if $\left|M\right|>0$ then29:    $M_{m-1}∥\cdots ∥M_{0}\leftarrow M$14:       $M_{m-1}∥\cdots ∥M_{0}\leftarrow M$30:    for i=0 to m−1 do15:       $t\leftarrow (\left|M_{m-1}\right|=n)$ ? 4 : 531:       $T\leftarrow {\tilde{E}}_{K}^{0}\left(T\right)$16:       T← FCBC${}^{*}$[$\tilde{E}$](K,T,M,t)32:       $C_{i}\leftarrow$ chop($T,\left|M_{i}\right|$) ⨁ $M_{i}$17:    return *T*33:    return ($C_{m-1}∥\cdots ∥C_{0}$)**Algorithm 2** sESTATE Encryption, Tag Creation, Authentication, and Decryption Algorithm [16]. 1: function ESTATE.ENC[$\tilde{E},\tilde{F}$](K,N,A,M)18: function ESTATE.DEC[$\tilde{E},\tilde{F}$](K,N,A,C,T)2:    $T\leftarrow MAC\left[\tilde{E},\tilde{F}\right]\left(K,N,A,M\right)$19:    $M\leftarrow OFB\left[\tilde{E}\right]\left(K,T,C\right)$3:    $C\leftarrow OFB\left[\tilde{E}\right]\left(K,T,M\right)$20:    $T^{\prime }\leftarrow MAC\left[\tilde{E},\tilde{F}\right]\left(K,N,A,M\right)$4:    retrun (C,T)21:    return $\left(T^{\prime }=T\right)$ ? *M* : ⊥ 5: function MAC[$\tilde{E},\tilde{F}$](K,N,A,M)22: function FCBC${}^{*}\left[\tilde{E},\tilde{F}\right]\left(K,T,D,t\right)$6:    if $\left|A\right|=0$ and $\left|M\right|=0$ then23:    $D_{d-1}∥\cdots ∥D_{0}\leftarrow D$7:       return $T\leftarrow {\tilde{E}}_{K}^{8}\left(N\right)$24:    for i=0 to d−2 do8:    $T\leftarrow {\tilde{F}}_{K}^{15}\left(N\right)$25:       $T\leftarrow {\tilde{F}}_{K}^{15}\left(T⊕D_{i}\right)$9:    if $\left|A\right|>0$ then26:    $T\leftarrow {\tilde{E}}_{K}^{t}\left(T⊕\mathrm{opt}\left(Dd-1\right)\right)$
10:       $A_{a-1}∥\cdots ∥A_{0}\leftarrow A$27:    return *T*11:       $t\leftarrow (\left|M\right|>0$ ; $\left|A_{a-1}\right|=n)$ ? 2 : 3 : 6 : 7
12:       T← FCBC${}^{*}\left[\tilde{E},\tilde{F}\right]\left(K,T,M,t\right)$28: function OFB[$\tilde{E}$](K,T,M)13:    if $\left|M\right|>0$ then29:    $M_{m-1}∥\cdots ∥M_{0}\leftarrow M$14:       $M_{m-1}∥\cdots ∥M_{0}\leftarrow M$30:    for i=0 to m−1 do15:       $t\leftarrow (\left|M_{m-1}\right|=n)$ ? 4 : 531:       $T\leftarrow {\tilde{E}}_{K}^{0}\left(T\right)$16:       T← FCBC${}^{*}$[$\tilde{E},\tilde{F}$](K,T,M,t)32:       $C_{i}\leftarrow$ chop($T,\left|M_{i}\right|$) ⨁ $M_{i}$17:    return *T*33:    return ($C_{m-1}∥\cdots ∥C_{0}$)

#### 2.5.1. TweAES-128, TweAES-128-6

Algorithm 3 are functions of TweAES-128, a cryptographic algorithm used in ESTATE mode. The overall process is the same as AES-128, and a process of XOR operation is added by expanding the 4-bit tweak value to an 8-bit tweak value for every even round except the last round. TweAES-128-6 is proposed and used as a cryptographic algorithm to be used while designing the sESTATE mode in the ESTATE algorithm. TweAES-128-6 has the same operation process as TweAES-128. The difference is that the TweAES-128 runs 10 rounds, while the TweAES-128-6 only runs 6 rounds.
**Algorithm 3** TweAES-128 Algorithm [16]. 1: function TweAES(K,T,M)15: function TweAES-6(K,T,X)2:    $(W_{43},...,W_{0})\leftarrow$ KeyGen(*K*)16: quad$(W_{43},...,W_{0})\leftarrow$ KeyGen(K,X)
3:    $X\leftarrow X⊕(W_{3},W_{2},W_{1},W_{0})$17:    $X\leftarrow X⊕(W_{3},W_{2},W_{1},W_{0})$4:    for i=1 to 9 do18:    for i=1 to 6 do5:       X← SubBytes(*X*)19:       X← SubBytes(*X*)6:       X← SubRows(*X*)20:       X← ShiftRows(*X*)7:       X← MixColumns(*X*)21:       X← MixColumns(*X*)8:       $X\leftarrow X⊕(W_{4i+3},W_{4i+2},W_{4i+1},W_{4i})$22:       $X\leftarrow X⊕(W_{4i+3},W_{4i+2},W_{4i+1},W_{4i})$9:       if i%2=0 then23:       if i%2=0 and i<6 then10:          X← AddTweak(X,T)24:          X← AddTweak(X,T)11:    X← SubBytes(*X*)25:    return *X*12:    X← ShiftRows(*X*)
13:    $X\leftarrow X⊕(W_{43},W_{42},W_{41},W_{40})$26: function AddTweak(X,T)14:    return *X*27:    $\left(X_{127},...,X_{0}\right)\overset{1}{\leftarrow }X$
28:    $\left(T_{3},...,T_{0}\right)\overset{1}{\leftarrow }T$
29:    $T_{⊕}\leftarrow T_{0}⊕T_{1}⊕T_{2}⊕T_{3}$
30:    for i=0 to 3 do
31:       $T_{i+4}\leftarrow T_{i}⊕T_{⊕}$
32:    for i=0 to 7 do
33:       $X_{8i}\leftarrow X_{8i}⊕T_{i}$
34:    return *X*

#### 2.5.2. TweGIFT-128

Algorithm 4 is the overall process of TweGIFT-128 used in ESTATE mode. TweGIFT-128 has the same structure as GIFT-128, and XOR operation is added by expanding the 4-bit tweak value to 32-bit tweak value every (round+1)%5 == 0th rounds. TweGIFT-128’s tweak expansion process is the same as TweAES-128. First, expand it to 8-bit in the same way, and then use the expanded 8-bit value to store the same value in the remaining 24-bits and expand it to a total of 32-bits.
**Algorithm 4** TweGIFT-128 Algorithm [16]. 1: function TweGIFT(K,T,X)11: function AddTweak(X,T)2:    C←00000012:    $\left(X_{127},...,X_{0}\right)\overset{1}{\leftarrow }X$3:    for i=0 to 39 do13:    $\left(T_{3},...,T_{0}\right)\overset{1}{\leftarrow }T$4:       X← SubCells(*X*)14:    $T_{⊕}\leftarrow T_{0}⊕T_{1}⊕T_{2}⊕T_{3}$5:       X← PermBits(*X*)15:    for i=0 to 3 do6:       (K,X)← AddRoundKey(K,X)16:       $T_{i+4}\leftarrow T_{i}⊕T_{⊕}$7:       (C,X)← AddRoundConstant(C,X)17:    $T_{15..8}\leftarrow T_{7..0}$8:       if (i+1)%5=0 and i<39 then18:    $T_{23..16}\leftarrow T_{7..0}$9:          X← AddTweak(X,T)19:    $T_{31..24}\leftarrow T_{7..0}$10:    return *X*20:    for i=0 to 31 do
21:       $X_{4i}\leftarrow X_{4i}⊕T_{i}$
22:    return *X*

## 3. Related Work

### 3.1. Existing Crypto Implementation Using OpenCL

Due to the development of multi-core processes, parallel processing technology is being used in various fields. In addition, the use of OpenCL for parallel processing is increasing, and it is efficient for processing large amounts of data. Therefore, studies are being conducted to rapidly encrypt a large amount of data using an cryptographic algorithm using OpenCL.

In Reference [22], we use OpenCL to improve encryption speed using the AES encryption algorithm. They used the NVIDIA GeForce GTX 1060 to measure performance. Table 3 is a table comparing the results measured in Reference [22] with previous studies. As a result, their research results show that the XTS (XEX-based tweaked-codebook mode with ciphertext stealing) mode is 12.86% and the CTR (Counter) mode is 14.71%, compared to the previous studies.

**Table 3:** AES (Advanced Encryption Standard) fast implementation study results comparison.

Paper	GPU	Language	Mode	Throughput (Gbps)
Yuan et al. [23]	ATI HD 7670M	OpenCL	CTR	5.04 Gbps
Wang et al. [24]	NVIDIA GTX 285	OpenCL	XTS	8.59 Gbps
Wang et al. [24]	NVIDIA GTX 285	CUDA	XTS	9.74 Gbps
Conti et al. [25]	NVIDIA GT 555M	OpenCL	CTR	10.00 Gbps
Biagio et al. [26]	NVIDIA GT 8800	CUDA	CTR	12.50 Gbps
Sanida et al. [22]	NVIDIA GTX 1060	OpenCL	XTS	12.53 Gbps
Sanida et al. [22]	NVIDIA GTX 1060	OpenCL	CTR	14.71 Gbps

In Reference [27], various cryptographic algorithms are implemented in OpenCL and used for image encryption. Table 4 is an information table that implements AES (Advanced Encryption Standard), DES (Data Encryption Standard), BlowFish, and RSA (Ron Rivest, Adi Shamir, Leonard Adleman) using OpenCL in Reference [27]. Table 5 is the result of measurement by CPU and GPU for each cryptographic algorithm implemented using OpenCL. As a result, AES, DES, BlowFish, and RSA show performance improvements of 8 times, 2.5 times, 11.13 times, and 5 times, respectively.

**Table 4:** Memory size, line of code for cryptographic algorithm implementation using OpenCL [27].

Cryptographic Algorithm	Key Size	Constant Space	Compilation Time
AES [20]	128-bit	844 KB	2.7 ms
DES [28]	192-bit	1294 KB	5.3 ms
BlowFish [29]	256-bit	252 B	3.5 ms
RSA [30]	128-bit	6 KB	1031 ms

In Reference [31], AES-256 encryption and decryption implementation using OpenCL parallel processing is compared with AES-256 implemented using sequential processing. As a result, when 10,240,000 work items are used, the implementation using OpenCL parallel processing shows performance improvement of about 240 times for encryption and 481 times for decryption. In addition, as measured by AMD Radeon HD 8850M and AMD Radeon HD 8570, AMD Radeon HD 8570 shows performance improvement of 3.8 times and 3.3 times in encryption and decryption, respectively.

### 3.2. Web Assembly

Web Assembly shows better performance than JavaScript in web-based applications, and due to continuous development, it will continue to be close to the performance of low-level languages, such as C language. In addition, research on Web Assembly is actively underway.

In Reference [6], the revised CHAM, *P*-256-wNAF (window Non-Adjacent Form), SHA-256 (Secure Hash Algorithm), and HMAC (Hash-based Message Authentication Code) algorithms are compared after implementation using Web Assembly and JavaScript for more efficient encryption, key exchange, and authentication in the web environment. Table 6 shows the performance measurement results for cryptographic algorithms, and it can be seen that it is more efficient when Web Assembly implements cryptographic algorithms than JavaScript. In addition, in the case of wNAF used for key exchange, the Atomic block method was applied to be safe from side-channel attack (SCA) [32]. Web Assembly shows that it can operate efficiently and safely because its performance overhead ratio is lower than that of JavaScript.

**Table 6:** Web Assembly and JavaScript performance measurement and comparison through cryptographic algorithm implementation (cpb: Cycle Per Byte) [6].

	Chrome		FireFox		Microsoft Edge	
	**Web Assembly**	**JavaScript**	**Web Assembly**	**JavaScript**	**Web Assembly**	**JavaScript**
revised CHAM-64/128 [33]	120 cpb (2.1 times)	260 cpb	120 cpb (2.1 times)	260 cpb	120 cpb (2 times)	240 cpb
revised CHAM-128/128 [33]	60 cpb (3 times)	180 cpb	60 cpb (1.6 times)	100 cpb	70 cpb (1.8 times)	130 cpb
revised CHAM-128/256 [33]	70 cpb (3 times)	210 cpb	70 cpb (2.1 times)	150 cpb	70 cpb (2.8 times)	200 cpb
wNAF	27 cpb (11 times)	300 cpb	30 cpb (12 times)	365 cpb	27 cpb (11 times)	322 cpb
wNAF [34] (Atomic block [35])	42 cpb (10 times)	447 cpb	37 cpb (10 times)	405 cpb	37 cpb (14 times)	522 cpb
wNAF (Improved Atomic block [6])	32 cpb (11 times)	365 cpb	32 cpb (12 times)	387 cpb	30 cpb (14 times)	437 cpb
SHA-256 [36]	27 cpb (7.5 times)	203 cpb	20 cpb (10.8 times)	216 cpb	20 cpb (11 times)	221 cpb
HMAC [37]	92 cpb (7.5 times)	697 cpb	93 cpb (24.8 times)	2315 cpb	97 cpb (7.1 times)	693 cpb

Reference [38] converts the Picnic algorithm [39] to Web Assembly, measures the performance in Chrome, FireFox, and Microsoft Edge, and compares it with the C/C++ implementation. As a result, the Picnic algorithm implemented by Web Assembly is about 2∼3 times slower than the C/C++ implementation.

### 3.3. Cache Timing Attack

There are various attack methods, such as differential attack and side-channel attack, to find out important information about encryption algorithm. In addition, there is an attack method that finds out the key value, which is important information of the cryptographic algorithm through the cache access time of the CPU, and research on this is being actively studied as interest in it increases. Ref. [40] proved the vulnerability through an attack to find the last round key against the T-table AES algorithm of OpenSSL 1.1.0f [41]. So, in Reference [40], they study and apply the T-table shuffling method to be safe against Flush + Reload, a kind of cache-timing attack [42]. In Reference [40], they randomly shuffle the array containing values from 0 to 255 using the Fisher-Yates function [43]. Then, the values stored in 4 256-byte T-tables are shuffled and used by using the shuffled array values. In Reference [40], the T-table was shuffled using the Fisher-Yates function in the AES T-table, and the test shows that it is safe against Flush + Reload cache-timing attacks.

## 4. Proposed Implementation for Secure Communication in Edge Computing Services

### 4.1. Overall Architecture of Proposed Software

The existing edge computing method processes data received from a server or user or data to be sent, and communicates through encryption and authentication. Therefore, in hardware, such as ARM, AVR, and RISC-V used in edge computing for encryption and authentication, secure communication is implemented by implementing encryption algorithms and authentication algorithms using programming languages suitable for each environment. However, since each environment uses different performance, different functions, and different programming languages, even the same algorithm needs to be implemented in each hardware. So, we use Web Assembly to implement encryption and authentication so that it can be used generally on each device. In addition, it uses the LWC ESTATE algorithm, which has both an encryption function and an authentication process. Web Assembly is designed for performance similar to a low-level language in a web environment. The ESTATE algorithm implemented by Web Assembly can be used in general without additional modification in PCs, smartphones, and IoT devices where web apps and web browsers can be used. Therefore, once created, it can be used in multiple devices for secure communication. In addition, the finally processed data is stored on the main server. Therefore, we propose additional optimization methods to use the ESTATE algorithm efficiently in the server. The operation process of the ESTATE algorithm has a characteristic that affects the next process using the previous value. Therefore, it is difficult to process a large amount of data at the same time. However, if the main server processes data sequentially, even if the edge computing method is used, the communication process eventually shows slow performance. So, we propose a method of using OpenCL parallel processing so that multiple ciphertexts and tags to be sent to multiple web-based applications can be created at the same time. In addition, to safely and efficiently operate the ESTATE algorithm, an additional method is proposed, and the previously existing studied methods are applied. During operation of TweAES-128, TweAES-128-6, and TweGIFT-128 used in the ESTATE algorithm, the 4-bit tweak value is checked for each specific round through conditional statements, and then expanded to perform XOR (exclusive OR) operation on the encrypted data. Therefore, we propose a method of storing and using the extended tweak values for 9 4-bit tweak values through pre-computation. So, tweak values are extended to 8-bit and 32-bit, respectively, through pre-computation. In the implementation of OpenCL, if there is a conditional statement, there is a load in the operation process. The ESTATE algorithm uses conditional statements due to the type of input value, tweak value check for each specific round, and tweak value XOR operation for each specific round. So, when we implement TweAES-128, TweAES-128-6, and TweGIFT-128 using OpenCL, we implement it using the loop unrolling method to eliminate performance degradation. In addition, it operates using local memory, which has a high operation speed. TweAES-128 and TweAES-128-6 are similar in operation to the AES algorithm. Therefore, it operates faster by applying the existing T-table method. In addition, there are studies that the AES algorithm is vulnerable to cache-timing attacks. Since TweAES-128 and TweAES-128-6, which have the same structure as the AES algorithm, can be vulnerable, they are safely operated by applying the T-table shuffling method, which is an the existing cache-timing attack response algorithm.

### 4.2. Edge Computing and Estate Implementation Using Web Assembly

**Figure 12:**
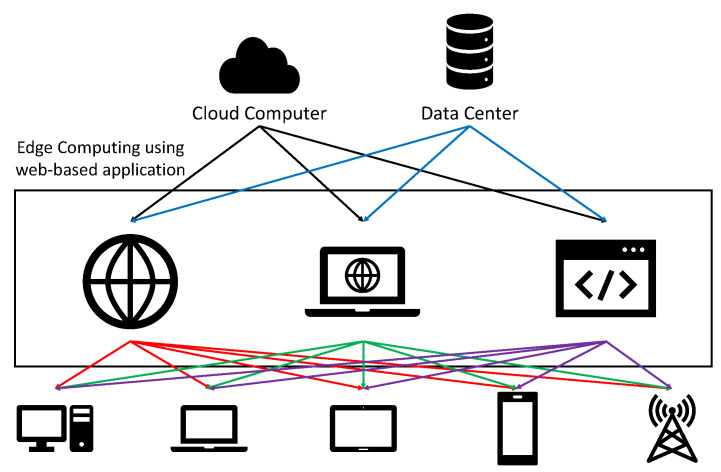
Edge computing structure using Web Assembly.

We propose a web-based application edge computing method using Web Assembly. Web Assembly was created to show performance similar to low-level language in web environment. The existing edge computing method provides services by optimizing each environment and functions in hardware, such as ARM, AVR, and RISC-V. However, this method is difficult to use in general because it uses programming languages and functions used in each environment, such as ARM, AVR, and RISC-V, and additional cost is consumed because additional implementation is required for each device. The web-based application edge computing method proposed by us can be used in PCs, smartphones, IoT devices, etc. that can basically use web-based applications. In addition, even if the platform is different, it is efficient because it can be used generally without additional modification in terms of implementation. In addition, in order to implement the algorithm with Web Assembly, the existing code implemented in a programming language with a data type can be converted and used, so there is no additional cost. In addition, if you use a library, such as Node.js, so that web socket communication is possible without adapting the communication process to each hardware, communication becomes easy. Web-based application In the edge computing method, communication between server and web-based application, communication between web-based application and user, and communication between web-based application are made. Encryption and authentication functions are required to safely send data in various communication processes. So, we use the ESTATE algorithm, which has encryption and authentication functions. Therefore, as shown in Figure 12, in a web-based application using Web Assembly, a ciphertext and a tag for authentication are created using the ESTATE algorithm, and data is safely delivered to the user.

### 4.3. Parallel Implementation of Estate Using OpenCL

The ESTATE algorithm uses TweAES-128 and TweGIFT-128 to encrypt each block of 128-bit size. Then, the next step is performed using the previously encrypted result value. Therefore, it is impossible to use a method of processing a large amount of data at once through parallel processing. It is designed for use in a limited environment, but the finally communicated data is stored on the server. Therefore, it is necessary to implement ESTATE according to the server environment so that the server can use ESTATE efficiently. We use OpenCL to simultaneously calculate and transmit ciphertext and tag generation to be sent to multiple web-based applications.

**Figure 13:**
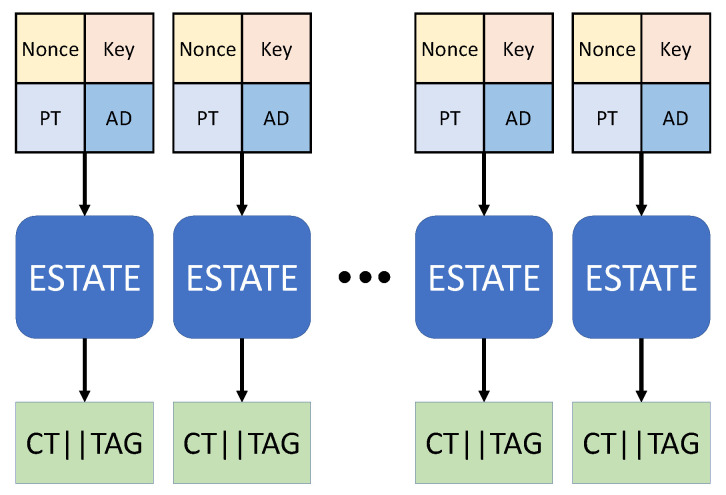
Structure of ESTATE algorithm operation using parallel processing.

Instead of sequentially processing multiple data using the ESTATE algorithm, it uses a method of simultaneously processing using OpenCL parallel processing as shown in Figure 13. When implemented using OpenCL parallel processing, performance degradation occurs when conditional statements exist. TweAES-128, TweAES-128-6, and TweGIFT-128 use conditional statements to check the type of input value, check whether or not padding, check the tweak value, and perform the extended tweak value XOR operation for each round. We use the loop unrolling method to remove the conditional statement in order to remove the performance load in the OpenCL implementation. In addition, the local memory has the fastest operation speed among OpenCL memories. For this reason, data is moved to local memory and encrypted to improve performance. Algorithm 5 is an OpenCL code algorithm that reduces the performance load by eliminating conditional statements using a loop unrolling method.
**Algorithm 5** TweAES-128, TweAES-128-6, TweGIFT-128 proposed by applying loop unrolling method. 1: function loop unrolling TweAES-128(K,T,X)41: function loop unrolling TweGIFT-128(K,T,X)2:    $(W_{43},...,W_{0})\leftarrow$ KeyGen(K,X)42:    C←0000003:    $X\leftarrow X⊕(W_{3},W_{2},W_{1},W_{0})$43:    for *i* = 0 to 7 do4:    for *i* = 1 to 4 do44:       for *j* = 0 to 3 do5:       X← SubBytes(*X*)45:          X← SubCells(*X*)6:       X← ShiftRows(*X*)46:          X← PermBits(*X*)7:       X← MixColumns(*X*)47:          (K,X)← AddRoundKey(K,X)8:       $X\leftarrow X⊕(W_{4i+3},W_{4i+2},W_{4i+1},W_{4i})$48:          (C,X)← AddRoundConstant(C,X) 9:        X← SubBytes(*X*)49:       X← SubCells(*X*)10:       X← ShiftRows(*X*)50:       X← PermBits(*X*)11:       X← MixColumns(*X*)51:       (K,X)← AddRoundKey(K,X)12:       $X\leftarrow X⊕(W_{8i+3},W_{8i+2},W_{8i+1},W_{8i})$52:       (C,X)← AddRoundConstant(C,X)13:       AddTweak(X, T)53:       AddTweak(X, T) 14:    X← SubBytes(*X*)54:    for *i* = 35 to 39 do15:    X← ShiftRows(*X*)55:       X← SubCells(*X*)16:    X← MixColumns(*X*)56:       X← PermBits(*X*)17:    $X\leftarrow X⊕(W_{39},W_{38},W_{37},W_{36})$57:       (K,X)← AddRoundKey(K,X)18:    X← SubBytes(*X*)58:       (C,X)← AddRoundConstant(C,X)19:    X← ShiftRows(*X*)
20:    $X\leftarrow X⊕(W_{43},W_{42},W_{41},W_{40})$
 21: function loop unrolling TweAES-6(K,T,X)
22:    $(W_{43},...,W_{0})\leftarrow$ KeyGen(K,X)
23:    $X\leftarrow X⊕(W_{3},W_{2},W_{1},W_{0})$
24:    for *i* = 1 to 2 do
25:       X← SubBytes(*X*)
26:       X← ShiftRows(*X*)
27:       X← MixColumns(*X*)
28:       $X\leftarrow X⊕(W_{4i+3},W_{4i+2},W_{4i+1},W_{4i})$
 29:       X← SubBytes(*X*)
30:       X← ShiftRows(*X*)
31:       X← MixColumns(*X*)
32:       $X\leftarrow X⊕(W_{8i+3},W_{8i+2},W_{8i+1},W_{8i})$
33:       AddTweak(X, T)
 34:    X← SubBytes(*X*)
35:    X← ShiftRows(*X*)
36:    X← MixColumns(*X*)
37:    $X\leftarrow X⊕(W_{23},W_{22},W_{21},W_{20})$
38:    X← SubBytes(*X*)
39:    X← ShiftRows(*X*)
40:    $X\leftarrow X⊕(W_{43},W_{42},W_{41},W_{40})$


### 4.4. Safe and Efficient Implementation of TweAES-128, TweAES-128-6, TweGIFT-128 of Estate Algorithm

TweAES-128 and TweGIFT-128 are used in ESTATE mode, and TweAES-128-6 is used in sESTATE mode. TweAES-128, TweGIFT-128, and TweAES-128-6 have the same operation process as AES-128 and GIFT-128, but additionally, the process of XOR operation by expanding the 4-bit tweak value is added. However, in TweAES-128, TweAES-128-6, and TweGIFT-128, only 0∼7, 15 are used as tweak values. Therefore, we propose a method to extend the 4-bit tweak value to 8-bit and 32-bit in advance to fit each algorithm and use it after storage. This method eliminates the unnecessary process of repeatedly checking and expanding tweak value. In addition, TweAES-128 and TweAES-128-6 have the same structure as the AES algorithm, so the existing studied T-table method to quickly compute AES can be applied. In addition, it is possible to perform faster operation by processing the 16-byte input value used in both algorithms in parallel.

**Figure 14:**
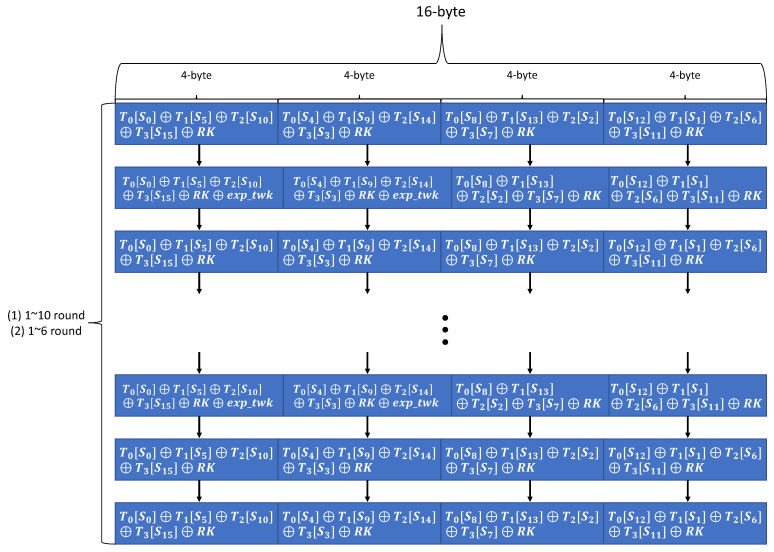
Parallel operation process of TweAES-128 or TweAES-128-6 using T-table method.

As shown in Figure 14, the operation process of TweAES-128 and TweAES-128-6 used in the ESTATE algorithm uses an efficient method of simultaneously calculating 16-byte input values through OpenCL parallel processing. In addition, T-table shuffling method, which is the method studied in Reference [40], is applied to the T-table used in ESTATE TweAES-128 and TweAES-128-6 to safely operate against cache-timing attack.

Using method in Reference [40], mix the index value of 0∼255 to shuffle the T-table. Then, the T-table is shuffled using the mixed index value. Algorithm 6 is a process that will be used every round of ESTATE TweAES-128 and TweAES-128-6.
**Algorithm 6** ESTATE TweAES-128, TweAES-128-6 Proposal Method Applying T-table Shuffling1: Te0-sf : Te0[shuffle-array]2: Te1-sf : Te1[shuffle-array]3: Te2-sf : Te2[shuffle-array]4: Te3-sf : Te3[shuffle-array] 5: function 1-round(S0∼S3, RK)6:    S0 = Te0-sf[S0 ≫ 24] ⊕ Te1-sf[S1 ≫ 16 & 0xff] ⊕ Te2-sf[S2 ≫ 8 & 0xff] ⊕ Te3-sf[S3 & 0xff] ⊕ RK7:    S1 = Te0-sf[S1 ≫ 24] ⊕ Te1-sf[S2 ≫ 16 & 0xff] ⊕ Te2-sf[S3 ≫ 8 & 0xff] ⊕ Te3-sf[S0 & 0xff] ⊕ RK8:    S0 = Te0-sf[S2 ≫ 24] ⊕ Te1-sf[S3 ≫ 16 & 0xff] ⊕ Te2-sf[S0 ≫ 8 & 0xff] ⊕ Te3-sf[S1 & 0xff] ⊕ RK9:    S0 = Te0-sf[S3 ≫ 24] ⊕ Te1-sf[S0 ≫ 16 & 0xff] ⊕ Te2-sf[S1 ≫ 8 & 0xff] ⊕ Te3-sf[S2 & 0xff] ⊕ RK 10: function 1-round with AddTweak(S0∼S3, RK, tweak)11:    S0 = Te0-sf[S0 ≫ 24] ⊕ Te1-sf[S1 ≫ 16 & 0xff] ⊕ Te2-sf[S2 ≫ 8 & 0xff] ⊕ Te3-sf[S3 & 0xff] ⊕ RK12:    S1 = Te0-sf[S1 ≫ 24] ⊕ Te1-sf[S2 ≫ 16 & 0xff] ⊕ Te2-sf[S3 ≫ 8 & 0xff] ⊕ Te3-sf[S0 & 0xff] ⊕ RK13:    S0 = Te0-sf[S2 ≫ 24] ⊕ Te1-sf[S3 ≫ 16 & 0xff] ⊕ Te2-sf[S0 ≫ 8 & 0xff] ⊕ Te3-sf[S1 & 0xff] ⊕ RK14:    S0 = Te0-sf[S3 ≫ 24] ⊕ Te1-sf[S0 ≫ 16 & 0xff] ⊕ Te2-sf[S1 ≫ 8 & 0xff] ⊕ Te3-sf[S2 & 0xff] ⊕ RK15:    AddTweak(S0∼S3, tweak)

## 5. Results

Table 7 is an environment in which the results were measured by applying the methods proposed by us to the ESTATE algorithm using OpenCL parallel processing, the ESTATE algorithm implemented with Web Assembly, and the reference C ESTATE algorithm.

Table 8 is a comparison result of OpenCL parallel processing, AES T-table, extended tweak pre-computation, and ESTATE algorithm applying loop unrolling methods and the reference C code ESTATE algorithm for sequential processing. We measured the process of creating a total of 6,400 ciphertexts and tags, respectively. As a result, in ESTATE TweAES-128, TweAES-128-6 and TweGIFT-128, OpenCL was 6.69 times, 7.31 times, and 1.47 times faster than the reference C/C++ code, respectively.

Table 9 shows the result of comparing the algorithm to which the T-table shuffling method was applied and the algorithm not applied. This is a measurement result of the process of shuffling and calculating the 1024-byte T-table. Due to shuffling, performance overhead occurs because memory must be accessed twice, unlike the method not applied. As a result, ESTATE TweAES-128 and TweAES-128-6 show performance overhead of 7% and 51%, respectively.

Table 10 shows how much performance overhead occurs compared to C language by implementing the ESTATE algorithm in Web Assembly to use the edge computing method using Web Assembly. Measurements were made for C and Web Assembly using the same input values. Web Assembly was measured on Chrome, FireFox, and Microsoft Edge. As a result, TweAES-128, TweAES-128-6, and TweGIFT-128 implemented as Web Assembly have 11%, 5%, 22% performance overhead in Chrome, 10%, 2%, 54 in FireFox. It shows % performance overhead, and 9%, 6%, and 17% performance overhead in Microsoft Edge. The reason the performance overhead ratio is different for each web browser is that the rendering engine and JavaScript engine used for each web browser are different. However, in the case of TweAES-128 and TweAES-128-6, the performance overhead is not large, so it can be seen that it is efficient to perform edge computing through a web-based application using Web Assembly.

## 6. Conclusions

The existing edge computing method takes over the role of cloud computing services in hardware, such as ARM, AVR, and RISC-V. Therefore, there is a disadvantage of having to implement separately using a function and programming language suitable for each environment used in ARM, AVR, and RISC-V. In this paper, we propose a web-based application edge computing method using Web Assembly in order to use an efficient edge computing method.

1 Implementation of ESTATE algorithm using Web Assembly
  Web Assembly was created to show similar performance to low-level language in a web environment. Cryptographic algorithms using web-based applications can use web-based applications, and can be used without additional modification in PCs, smart phones, and IoT devices used as edge devices. Therefore, even if the platforms used are different, it is also cost-effective because it can be used generally without additional modification in terms of implementation. In addition, web-based application edge computing communicates with various platforms, so, to send data securely, we implement and use the ESTATE algorithm, which has both encryption and authentication processes, in Web Assembly. We can see how Web Assembly has caught up with the performance of low-level languages. ESTATE Web Assembly implementation compares performance with reference C/C++ code. Web Assembly implementation is measured in web browsers Chrome, FireFox, and Microsoft Edge. As a result, TweAES-128, TweAES-128-6, and TweGIFT-128 implemented as Web Assembly have 11%, 5%, 22% performance overhead in Chrome, 10%, 2%, 54 in FireFox. It shows % performance overhead, and 9%, 6%, and 17% performance overhead in Microsoft Edge. As a result, it is slower than C/C++, which is a low-level language, but it can be used efficiently because it can be used without special modifications on devices that can use web-based applications.
2 ESTATE algorithm using OpenCL parallel processing
  Data processed by the web-based application edge computing method are eventually stored on the main server. Therefore, in order to use the ESTATE algorithm efficiently, it is necessary to implement it according to the server environment. So, we propose a method of simultaneously processing ciphertext and tag generation to be sent to multiple platforms using OpenCL parallel processing. Through OpenCL parallel processing, each byte value is processed simultaneously instead of sequentially for the 16-byte input value used for one encryption process. OpenCL has a load when using conditional statements. In the ESTATE algorithm, a conditional statement is used to XOR the extended tweak value every specific round. Therefore, the loop unrolling method was used to remove the performance load by removing the process of using conditional statements. In addition, data is stored in a local memory with a fast operation speed and encrypted to perform efficient operation. For performance comparison, we compare the OpenCL parallel processing implementation and the reference C/C++ sequential processing implementation. As a result, the OpenCL implementation shows about 6.69 times, 7.31 times, and 1.47 times performance improvement in ESTATE TweAES-128, TweAES-128-6, and TweGIFT-128 than the reference C/C++ implementation.
3 Method for efficient and safe operation of ESTATE algorithm
  Additional methods are applied to safely and efficiently operate the ESTATE algorithm itself. The ESTATE algorithm uses conditional statements to check the type of input value to be encrypted, check whether it is the last block, check the tweak value, and calculate the extended tweak value for each specific round. The 8-bit and 32-bit extended tweak values used in TweAES-128, TweAES-128-6, and TweGIFT-128 are stored and used in advance through pre-calculation. This method reduces the performance load by removing unnecessary conditional statements. In addition, TweAES-128 and TweAES-128-6 have the same operation process as the AES algorithm, so they may be vulnerable to cache-timing attacks. So, we apply the T-table shuffling method, which is a previously studied method, to operate safely. We reduced the performance load by applying the proposed methods to minimize the performance load even when the T-table shuffling method is applied. As a result of applying the T-table shuffling method, TweAES-128 and TweAES-128-6 show about 7% and 51% performance overhead, respectively, than those without applying the T-table shuffling method.
4 Future Work
  Web-based application using Web Assembly can be used in various devices without additional modification, so it can reduce the system load of the server and is effective in responding to failures. Web Assembly is currently continuously developing, and, since various devices, such as PCs, smart phones, and smart devices, are developing more and more, web technology is also developing accordingly. Currently, technologies using high-end hardware, such as Web Assembly’s SIMD technology and WebGPU, are being developed. In addition, it is being developed so that Web Assembly and WebGPU can be used together. When these technologies become stable in the future, many web developers will develop web services using various technologies, such as SIMD and WebGPU. Therefore, it can be used in various ways in terms of crypto security, and various studies will be conducted using web technologies developed in the field of crypto security. Therefore, the web-based application edge computing method can also be developed, and performance will be improved. Currently, there are various NIST LWC (National Institute of Standards and Technology LightWeight Cryptography) Round 2 candidate algorithms. However, the OpenCL parallel processing method we used is a method applicable to other candidate algorithms. Even if the LWC algorithm other than ESTATE is used to send data to multiple devices, the service can be provided more efficiently by using the method of simultaneously processing multiple ciphertexts and tags through the OpenCL parallel processing method.

## Figures and Tables

**Table 2:** Energy efficient and Single-state Tweakable block cipher-based MAC-Then-Encrypt (ESTATE) notation [16].

Notation	Denote	Notation	Denote
$\left|A\right|$	length(bit) of A	*K*	$K\in {\left\{0,1\right\}}^{k}$
$\left(X_{k-1},...,X_{0}\right)\overset{8}{\leftarrow }x$	n-bit block parsing of X	*T*	$T\in {\left\{0,1\right\}}^{t}$
*i*	0≤i≤k−2	*M*	$M\in {\left\{0,1\right\}}^{m}$
$\left|X_{i}\right|$	$\left|X_{i}\right|=n$	$\tilde{E}$	TweAES-128 or TweGIFT-128
$\left|X_{k-1}\right|$	$1\leq \left|X_{k-1}\right|\leq n$	$\tilde{F}$	TweAES-128-6
A⊕B	the bitwise XOR of A and B	X⋘i	*i*-bit left
A||B	the concatenation of A and B	X⋙i	*i*-bit right
n	128-bit block size	k	128-bit key size
t	128-bit tag size	τ	4-bit tweak size

**Table 5:** Measuring cryptographic algorithm results using OpenCL [27].

Device	AES	DES	BlowFish	RSA
AMD FX 6100 3.0 GHz (CPU 6 Cores)	240 Mbps	144 Mbps	736 Mbps	4 Mbps
NVIDIA GTX 550 (GPU)	1920 Mbps	368 Mbps	8192 Mbps	20 Mbps
Ratio of Performance Improvement	8 times	2.5 times	11.13 times	5 times

**Table 7:** Performance measurement environment.

Operationg System	CPU	RAM	SW	Languages and API	Used Input Value	ESTATE Operation Count
Window 10 Education	Intel i5-8250U 1.6 GHz	8 GB	(1) Chrome (2) FireFox (3) Microsoft Edge	(1) C/C++ (2) Web Assembly (3) OpenCL	Nonce: 25,600-byte AD: 51,200-byte Message: 512,000-byte	6400

**Table 8:** Performance comparison of OpenCL implementation and C/C++ reference code applying the proposed method (ns: nanosecond).

Algorithm	OpenCL	Reference C/C++	Performance Improvement
ESTATE TweAES-128	19,088,500 ns	127,842,877 ns	6.69 times
ESTATE TweAES-128-6	15,966,333 ns	116,813,270 ns	7.31 times
ESTATE TweGIFT-128	1,958,343,000 ms	2,897,251,400 ns	1.47 times

**Table 9:** Performance overhead measurement result through application of T-table shuffling method (ns: nanosecond).

Algorithm	Applied T-Table Shuffling Method	Normal Method	Performance Overhead
ESTATE TweAES-128	20,589,394 ns	19,088,500 ns	7%
ESTATE TweAES-128-6	24,192,899 ns	15,966,333 ns	51%

**Table 10:** Performance overhead measurement result of ESTATE algorithm using Web Assembly (ns: nanosecond).

Algorithm	Reference C/C++ Code	Web Assembly		
		Chrome (Performance Overhead)	FireFox (Performance Overhead)	Microsoft Edge (Performance Overhead)
ESTATE TweAES-128	127,842,877 ns	142,775,000 ns (11%)	141,000,000 ns (10%)	140,374,999 ns (9%)
ESTATE TweAES-128-6	116,813,270 ns	123,155,001 ns (5%)	120,000,000 ns (2%)	124,045,001 ns (6%)
ESTATE TweGIFT-128	2,897,251,400 ns	3,560,440,001 ns (22%)	4,490,000,000 ns (54%)	3,401,205,000 ns (17%)

## Data Availability

Not applicable.
